# Pathological mechanisms and clinical research progress of endothelial dysfunction

**DOI:** 10.3389/fcvm.2026.1749548

**Published:** 2026-02-04

**Authors:** Zhuoran Wang, Yuqiao Yang, Quan Wang, Lingyan Wang, Yu Zhao, Xi Qian, Rui Feng, Jinqiao Qian

**Affiliations:** Department of Anesthesiology, The First Affiliated Hospital of Kunming Medical University, Kunming, Yunnan, China

**Keywords:** biomarkers, clinical research, endothelial dysfunction, oxidative stress, pathological mechanisms, treatment progress

## Abstract

Endothelial dysfunction (ED) has emerged as a critical pathological contributor to a variety of cardiovascular and metabolic disorders, garnering increasing attention in recent years. This review presents a comprehensive overview of ED, commencing with its definition and broadened criteria. It delves into the molecular mechanisms underlying ED, including reduced nitric oxide availability, oxidative stress, inflammatory responses, apoptosis of endothelial cells, and the emerging concept of endothelial-mesenchymal transition. Furthermore, we investigate the association between ED and multiple conditions, such as diabetes, atherosclerosis, cerebrovascular disorders, and obstructive sleep apnea, drawing on finding from recent clinical investigations. The review highlights the clinical significance and limitations of various biomarkers associated with ED. Moreover, we explore contemporary pharmacological treatment modalities and pioneering therapeutic strategies to alleviate ED, including small molecule agents, stem cell therapy, and gene therapy. By integrating the most recent discoveries from both fundamental and clinical research, this review aims to establish a robust theoretical framework and provide practical guidelines for the diagnosis, prevention, and management of ED.

## 1 Introduction

Endothelial dysfunction (ED) is a significant pathological condition that serves as a precursor to various cardiovascular diseases, metabolic syndromes, and neurological disorders. This condition is characterized by impaired homeostatic functions of endothelial cells, which are crucial for regulating vascular tone, permeability, and inflammatory responses. The endothelium, a single layer of cells that lines blood vessels, is essential for maintaining vascular health and responding to hemodynamic changes. Various factors, including oxidative stress, inflammation, and metabolic disturbances, can trigger ED, which in turn contributes to conditions such as atherosclerosis, hypertension, and other cardiovascular complications (1).

Recent research has highlighted the complex role of endothelial cells in maintaining vascular homeostasis. These cells respond dynamically to stimuli such as shear stress, which is crucial for preserving the structural integrity of blood vessels. Rather than serving as mere passive barriers, endothelial cells actively engage in signaling pathways that regulate vascular function and adapt to pathological conditions. This underscores the importance of understanding the mechanisms that lead to ED, as this dysfunction is closely associated with the development and progression of cardiovascular diseases (2).

The traditional definition of ED has expanded to include a broader spectrum of criteria beyond impaired vasodilation and increased permeability. It now encompasses mechanisms like endothelial-to-mesenchymal transition (EndMT), a process in which endothelial cells lose their typical characteristics and gain mesenchymal traits. This transition plays a significant role in vascular remodeling and fibrosis. Such changes are especially relevant in chronic inflammatory conditions, such as those associated with diabetes and obesity, where ongoing ED can result in serious cardiovascular consequences (3).

The connection between ED and systemic diseases is gaining increasing recognition. For example, in cases of metabolic syndrome, the interaction between ED and inflammation related to obesity significantly contributes to cardiovascular risk. Patients often exhibit increased levels of pro-inflammatory cytokines and markers of oxidative stress, which further exacerbate their condition (4). Recognizing these relationships is essential for creating effective treatment strategies that focus on restoring endothelial function and preventing related complications.

ED has significant clinical implications, as it is associated with higher rates of morbidity and mortality from cardiovascular events. Therefore, identifying reliable biomarkers for ED is essential for early detection and timely intervention. Current research is exploring a range of biomarkers, such as soluble adhesion molecules, inflammatory cytokines, and circulating endothelial microparticles, which may provide valuable insights into the endothelial health of patients (5).

In summary, ED is a complex condition influenced by multiple factors, significantly contributing to the development of cardiovascular diseases, metabolic syndromes, and neurological disorders. Gaining a deeper understanding of the mechanisms behind ED is crucial for creating targeted therapies aimed at improving vascular health and reducing the associated risks of these conditions. The increasing prevalence of related diseases highlights the urgent need for research in this area, as effective management strategies are essential for enhancing patient outcomes.

## 2 Main body

### 2.1 Definition and pathological criteria of ED

#### 2.1.1 Traditional definition: diminished vascular relaxation function and pro-inflammatory, pro-coagulation state

ED is primarily characterized by impaired vascular relaxation, largely due to reduced bioavailability of nitric oxide (NO), accompanied by a pro-inflammatory and pro-thrombotic state. NO plays a vital role in maintaining vascular homeostasis, and when its production is insufficient, it can hinder the ability of blood vessels to dilate properly. Research indicates that conditions such as obesity and metabolic disorders can significantly reduce the availability of NO, worsening ED and increasing the risk of vascular complications (6). Moreover, when exposed to inflammatory signals, endothelial cells often become activated, which leads to a rise in the expression of adhesion molecules and pro-inflammatory cytokines. This activation is linked to the upregulation of nicotinamide adenine dinucleotide phosphate (NADPH) oxidase and other pathways that generate reactive oxygen species (ROS), further intensifying oxidative stress and damaging the endothelium (6, 7). The resulting inflammatory milieu not only disrupts vasodilation but also fosters a pro-coagulation state, marked by heightened platelet activation and increased expression of coagulation factors, which ultimately predisposes individuals to thrombotic events (8, 9).

Endothelial permeability also undergoes significant alterations during dysfunction, resulting in increased vascular leakage and inflammation. This enhanced permeability is frequently driven by inflammatory cytokines, which can compromise the endothelial glycocalyx (a critical barrier preventing leukocyte adhesion and extravasation) (10). The relationship between inflammation and coagulation becomes particularly pronounced in conditions like sepsis, where the activation of the NOD-, LRR-, and pyrin domain-containing protein 3 (NLRP3) inflammasome has been linked to the promotion of ED through mechanisms that involve the proteolysis of endothelial nitric oxide synthase (eNOS) (7). This dysregulation of pathways underscores the intricate connection between endothelial function and systemic inflammation, highlighting the necessity for therapeutic approaches that address both vascular relaxation and inflammatory responses to alleviate the detrimental effects of ED.

In summary, the traditional definition of ED involves a complex interaction characterized by reduced capacity for vascular relaxation and a state that promotes inflammation and coagulation. The decrease in nitric oxide availability, along with the activation of endothelial cells and heightened permeability, fosters a pathological environment that increases the risk of cardiovascular complications. Grasping these mechanisms is crucial for creating targeted interventions that aim to restore endothelial function and prevent the advancement of associated diseases.

#### 2.1.2 Expanded definition: mechanical signal transduction impairment and EndMT

The endothelial cells, which serve as a vital barrier between blood and tissues, possess an inherent ability to detect mechanical stimuli from their surroundings, a phenomenon referred to as mechanotransduction. This capability is crucial for maintaining vascular homeostasis and adapting to physiological demands. However, recent research suggests that the process of mechanical signal transduction can be disrupted under pathological conditions, leading to ED. For example, in cardiovascular diseases, changes in shear stress and heightened mechanical load can interfere with normal mechanotransduction pathways, initiating abnormal signaling cascades that activate and impair endothelial cells (11). The reduction in endothelial mechanosensitivity can worsen conditions like atherosclerosis, where the inadequate response to mechanical forces increases vascular permeability and inflammation (12). As endothelial cells lose their ability to sense mechanical changes, they may undergo EndMT, acquiring mesenchymal traits, such as enhanced motility and resistance to cell death, while losing their typical endothelial features (13). This transition is not merely a phenotypic shift, it has significant consequences for tissue remodeling and fibrosis, especially in chronic inflammatory conditions and during the healing of wounds (14).

The pathological significance of EndMT is considerable, as it contributes to diverse diseases, including fibrosis, cancer metastasis, and vascular disorders. This transition involves endothelial cells changing into a mesenchymal phenotype, often driven by factors like transforming growth factor-beta (TGF-β). TGF-β activates signaling pathways that encourage the expression of mesenchymal markers while suppressing endothelial markers (15). This process is especially pertinent in diabetes, where hyperglycemia-induced oxidative stress can trigger EndMT, leading to vascular complications associated with the condition (16). Additionally, the mechanical environment can significantly affect EndMT; for instance, heightened mechanical stress from hypertension can increase the expression of TGF-β and other fibrotic markers, thereby facilitating the transition to a mesenchymal phenotype (17).

In summary, impaired mechanotransduction in endothelial cells plays a pivotal role in ED and the ensuing EndMT process. This transition not only changes the cellular phenotype but also contributes to various pathological conditions. Elucidating the mechanisms underlying mechanotransduction and EndMT is essential for developing targeted therapeutic strategies for vascular diseases and related disorders. The interaction between mechanical signals, cellular responses, and pathological outcomes underscores the complexity of endothelial biology and its relevance.

#### 2.1.3 *In vivo* and *in vitro* models and their contributions to the definition of ED

The study of ED has advanced substantially through the development of diverse *in vivo* and *in vitro* models that recapitulate relevant pathophysiological conditions. Notably, models that simulate the blood-brain barrier have been instrumental in elucidating the mechanisms behind ED. For example, researchers have utilized human-induced pluripotent stem cell (iPSC)-derived endothelial cells to reproduce the diabetic ED phenotype *in vitro*, clearly demonstrating how hyperglycemia impairs endothelial cell functionality (18). Moreover, incorporating pulsatile flow into endothelial cell cultures enhances their physiological relevance of these models, as it more accurately reflects the hemodynamic forces encountered *in vivo*. This method has shed light on the role of altered mechanotransduction in ED, emphasizing how shear stress affects endothelial cell behavior and integrity. Additionally, endothelial lysates obtained from animal models have enabled gene and protein profiling, offering valuable insights into the molecular changes associated with ED (19). These models not only deepen our understanding of the underlying pathophysiological mechanisms but facilitate identification of potential biomarkers and therapeutic targets for ED.

Recent advancements in modeling techniques have significantly enhanced our understanding of ED in various neurological conditions, particularly in the context of the blood-brain barrier. For instance, the use of microfluidic platforms that mimic the blood-brain barrier models has facilitated the investigation of endothelial permeability and the impact of inflammatory mediators in a controlled setting (20). These innovative models have revealed that hypoxia, which is commonly associated with numerous diseases, exacerbates ED by inducing upregulation of enzymes such as arginase II thereby disrupting intercellular junctions, leading to increased permeability (21). Additionally, the integration of dynamic flow conditions into these models has shed light on the mechanistic pathways that contribute to heightened endothelial permeability and dysfunction, especially in relation to aging and neurodegenerative diseases (22).

The use of *in vivo* models, particularly those involving diabetic mice, has played a crucial role in confirming the results obtained from *in vitro* studies. These models have demonstrated the harmful effects of high blood sugar levels on endothelial function, showing that endothelial cells from diabetic models have reduced ability to form new blood vessels and exhibit heightened inflammatory responses (23). This underscores the importance of combining *in vitro* and *in vivo* approaches to capture the full complexity of ED, as each type of model brings distinct advantages that enhance our overall comprehension. By integrating these approaches, researchers have gained a more complete insight into the underlying mechanisms of ED, which is essential for developing targeted treatments aimed at restoring endothelial function in those affected.

Examples of widely used *in vivo* ED models include streptozotocin-induced or db/db diabetic mice (microvascular ED), ApoE^−/−^ or LDLR^−/−^ mice fed a high-fat/high-cholesterol diet (atherosclerosis-associated ED), chronic angiotensin II infusion or L-NG-nitroarginine methyl ester administration (hypertension-related ED), cecal ligation and puncture or lipopolysaccharide (LPS) endotoxemia (sepsis-associated ED), and chronic intermittent hypoxia to model obstructive sleep apnea -related ED. Complementary *in vitro* paradigms include human umbilical vein endothelial cell or human aortic endothelial cell exposure to high glucose, oxidized low-density lipoprotein (OxLDL), or cytokines and shear-stress microfluidic systems to recapitulate mechanotransduction defects (22, 24–28).

In conclusion, advancements in both *in vivo* and *in vitro* models have greatly enhanced our understanding of ED. These models have played a crucial role in exploring the underlying mechanisms and have offered valuable insights into potential therapeutic interventions. As research progresses, incorporating innovative modeling strategies will further deepen our understanding of ED and its implications for various cardiovascular and neurological diseases.

### 2.2 Molecular mechanisms of ED

#### 2.2.1 Integrative molecular cascades and cross-talk among NO-ROS, inflammation, and EndMT

**Figure 1:**
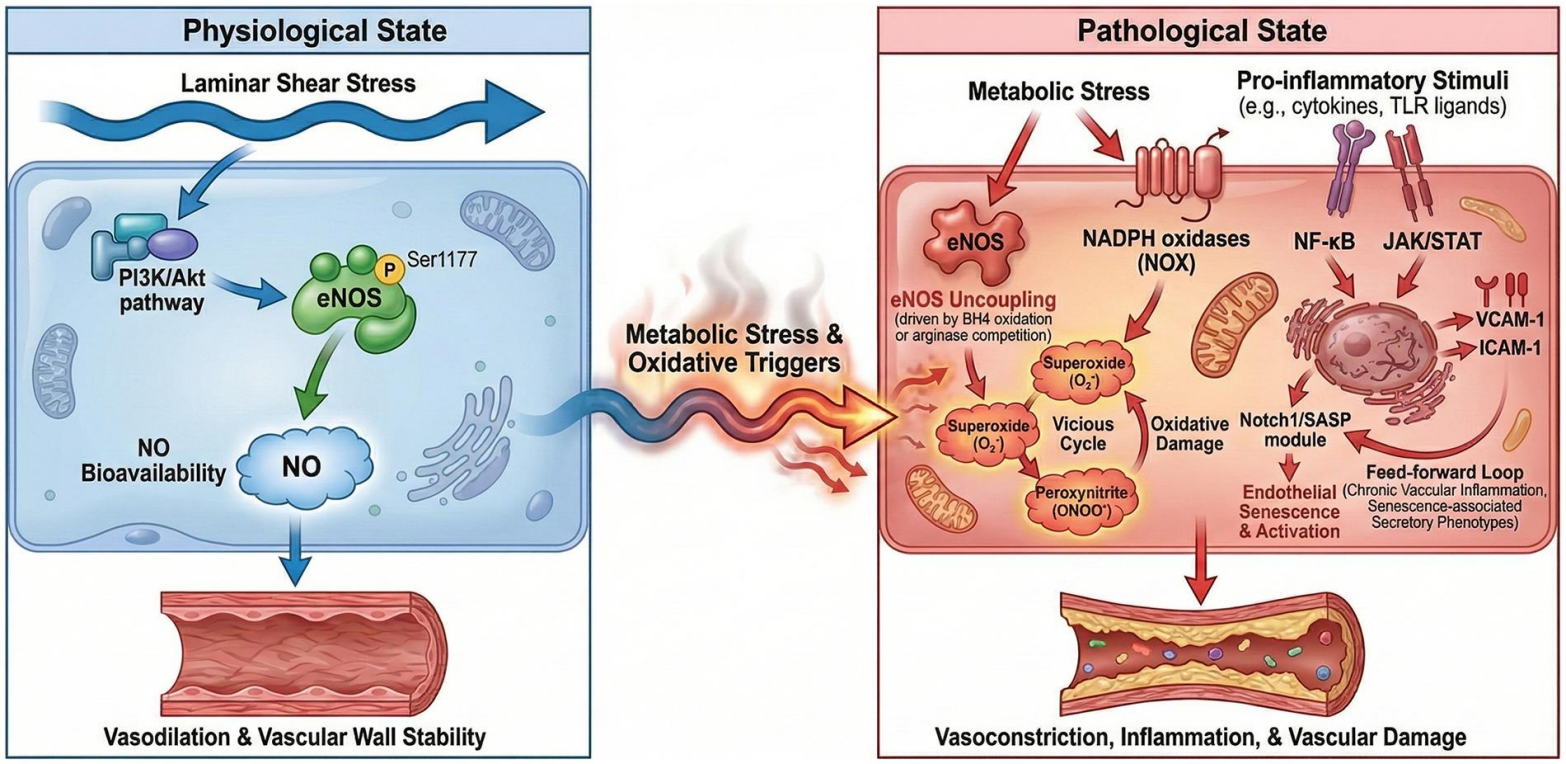
Integrative network of the NO-ROS axis and inflammatory signaling in endothelial dysfunction. Figure 1 illustrates the transition from physiological homeostasis to a pathological state driven by metabolic stress and inflammatory activation. Physiological state (left): under normal laminar shear stress, the PI3K/Akt pathway maintains eNOS phosphorylation at Ser1177, ensuring steady nitric oxide (NO) bioavailability, which promotes vasodilation and vascular wall stability. Pathological transition (right): metabolic stress and oxidative triggers induce eNOS uncoupling (driven by BH4 oxidation or arginase competition) and activate NADPH oxidases (NOX), shifting the balance toward superoxide and peroxynitrite formation. This “vicious cycle” impairs NO signaling and facilitates oxidative damage. Inflammatory integration: pro-inflammatory stimuli (e.g., cytokines, TLR ligands) activate NF-*κ*B and JAK/STAT pathways, orchestrating the upregulation of adhesion molecules (VCAM-1, ICAM-1). Additionally, the Notch1/SASP module drives endothelial senescence and activation, creating a feed-forward loop that sustains chronic vascular inflammation and senescence-associated secretory phenotypes.

**Figure 2:**
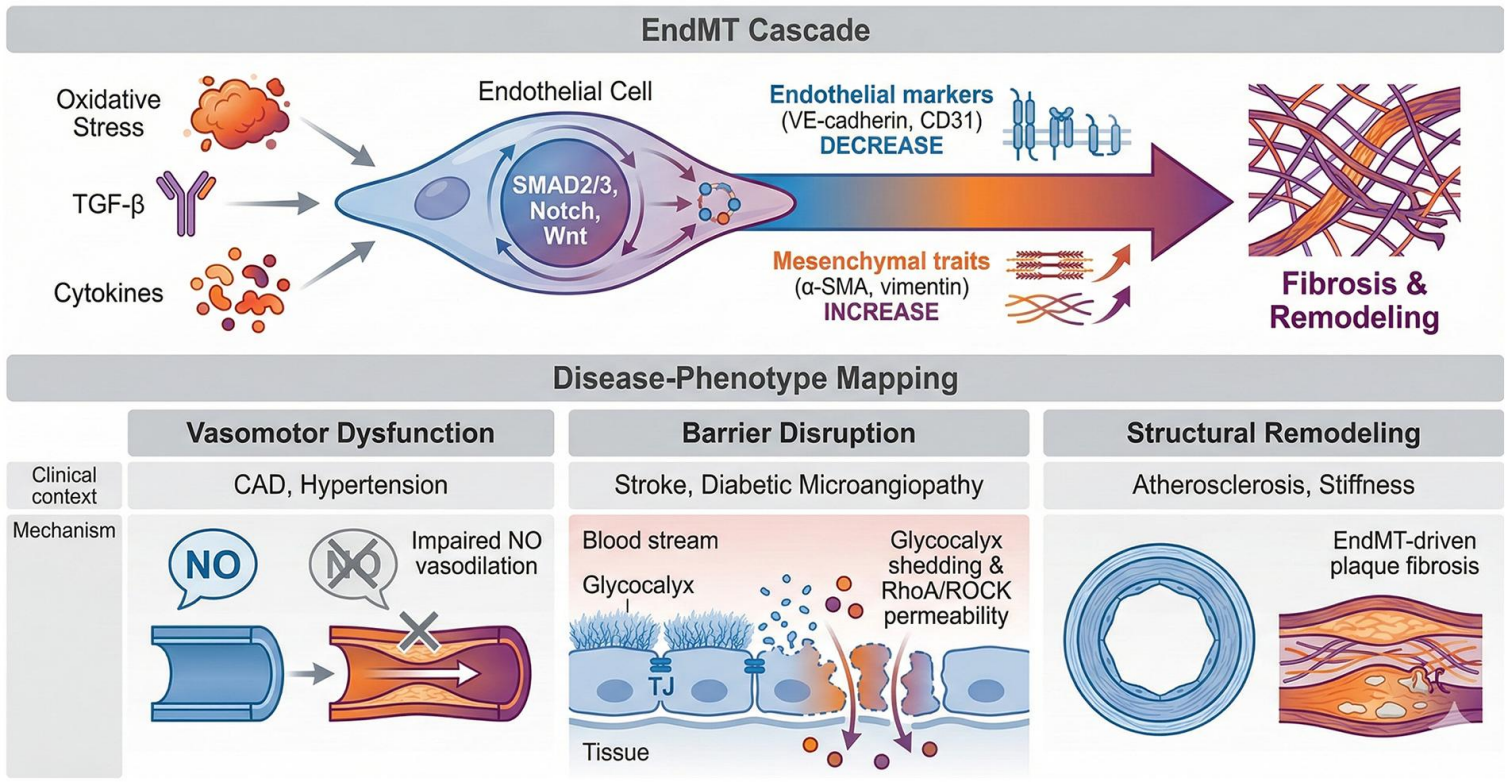
The EndMT cascade and its mapping to disease-specific endothelial phenotypes. Figure 2 delineates the molecular trajectory of endothelial-to-mesenchymal transition (EndMT) and characterizes the dominant endothelial dysfunction (ED) phenotypes across different clinical contexts. EndMT cascade (upper): chronic oxidative stress, TGF-β, and inflammatory cytokines (IL-1β, TNF-α) act as the “pathological bridge,” triggering SMAD2/3-dependent signaling and cooperating pathways (Notch, Wnt). This leads to the loss of endothelial markers (VE-cadherin, CD31) and the acquisition of mesenchymal traits (α-SMA, vimentin), ultimately resulting in structural vascular remodeling and fibrosis. Disease-phenotype mapping (lower): the diverse manifestations of ED are categorized into actionable phenotypes: (1). Vasomotor dysfunction: primarily observed in CAD, INOCA, and hypertension, characterized by impaired NO-mediated vasodilation. (2). Barrier disruption: noted in stroke and diabetic microangiopathy, involving glycocalyx shedding and RhoA/ROCK-mediated permeability. (3). Structural remodeling: Driven by EndMT and plaque fibrosis, contributing to advanced atherosclerosis and organ-specific vascular stiffness.

To move beyond a descriptive definition of ED, we outline below an explicit, directional network that links the NO-ROS axis, inflammatory signaling, and EndMT. This integrative view helps interpret disease-specific ED phenotypes and prioritize biomarkers and therapeutic targets (Figures 1, 2; Table 1).

**Table 1:** Major endothelial dysfunction phenotypes: core molecular cascades, representative readouts, and typical disease contexts.

Dominant ED phenotype	Core molecular cascade (key nodes)	Representative readouts	Typical disease contexts	Key references
Impaired vasodilation/NO deficiency	↓PI3K/Akt-eNOS, ↑ADMA/arginase, BH4 oxidation, eNOS uncoupling, ↑NOX/mitochondrial ROS	FMD/reactive hyperemia index; NO metabolites, ADMA; 3-NT; eNOS phosphorylation	Diabetes, obesity, INOCA/microvascular angina, hypertension	(50–55, 81–83, 100, 101)
Barrier disruption/increased permeability	Glycocalyx shedding, junctional protein loss, RhoA/ROCK activation, inflammatory cytokines, ROS	Endothelial EVs/microparticles; permeability imaging; albumin leakage; BBB metrics	Diabetic microangiopathy, acute ischemic stroke/BBB injury, OSA (intermittent hypoxia)	(10, 21–23, 26, 27, 104–108)
Inflammatory endothelial activation	NF-kB and JAK/STAT activation; ↑VCAM-1/ICAM-1/E-selectin; chemokines (MCP-1)	Soluble VCAM-1/ICAM-1, CRP/IL-6; leukocyte adhesion signatures	Atherosclerosis, metabolic syndrome, OSA, systemic inflammation	(24, 63, 65–68, 110, 111, 147)
Prothrombotic remodeling	↓NO/prostacyclin; ↑tissue factor; platelet activation; thromboinflammation modules	Platelet activation markers; coagulation panels (context-dependent)	CAD/ACS risk states, diabetes, severe inflammatory states	(8, 9)
EndMT/fibrotic remodeling	TGF-*β*-SMAD2/3 with Notch/Wnt cooperation; SNAIL/SLUG, ZEB/TWIST; ↑ECM	↓CD31/VE-cadherin, ↑*α*-SMA/vimentin; fibrosis markers; remodeling imaging	Diabetes-related vasculopathy, plaque fibrosis, PAH, organ fibrosis	(15–17, 72–80)

Key references correspond to the numbered reference list in the manuscript.

NO-ROS axis: physiological shear stress activates phosphoinositide 3-kinase/protein kinase B (PI3K/Akt) and eNOS (Ser1177 phosphorylation), increasing NO that signals via soluble guanylate cyclase/cyclic guanosine monophosphate to promote vasodilation and antithrombotic, anti-inflammatory programs (29–31). Under metabolic or inflammatory stress, NO bioavailability declines through (i) eNOS uncoupling driven by tetrahydrobiopterin (BH4) oxidation, (ii) increased superoxide (O_2_^•−^) generation from NADPH oxidases (NOX), mitochondria, and uncoupled eNOS itself, and (iii) substrate competition by asymmetric dimethylarginine (ADMA) or arginase II that limits L-arginine (32). Superoxide rapidly consumes NO to form peroxynitrite (ONOO−), which nitrates proteins (3-nitrotyrosine) and further impairs eNOS, creating a self-amplifying vicious cycle.

Mechanistic note on eNOS phosphorylation and coupling: Endothelial NO production is regulated not only by total eNOS expression but also by post-translational control of enzymatic coupling. Phosphorylation of eNOS at Ser1177 (human; Ser1179 in bovine) by Akt, AMPK, and PKA enhances electron flux and NO output, whereas phosphorylation at Thr495 and other inhibitory modifications reduce calmodulin binding and enzymatic activity. Full NO-generating activity also requires adequate L-arginine and cofactors such as tetrahydrobiopterin (BH4); BH4 oxidation or substrate limitation (e.g., ADMA/arginase competition) promotes eNOS uncoupling, shifting eNOS from NO generation to superoxide production and thereby amplifying oxidative stress and endothelial dysfunction (29, 33–35).

Major ROS sources and NO consumption: In dysfunctional endothelium, superoxide (O_2_^•−^) is produced predominantly by NADPH oxidases (NOX1/2/4/5), mitochondria, and uncoupled eNOS (and, in some contexts, xanthine oxidase). Superoxide rapidly reacts with NO to form peroxynitrite (ONOO^−^), which decreases NO bioavailability and drives oxidative/nitrative injury through protein tyrosine nitration (e.g., 3-nitrotyrosine) and oxidation of BH4. These modifications further impair eNOS coupling and endothelial barrier/vasomotor functions, constituting a self-amplifying NO–ROS vicious cycle (36, 37).

Inflammatory integration: cytokine and pattern-recognition receptor signaling [e.g., tumor necrosis factor-alpha (TNF-α)/ interleukin-1β(IL-1β) receptors, toll-like receptors (TLR)] activates nuclear factor kappa B (NF-*κ*B) and Janus kinase/signal transducer and activator of transcription (JAK/STAT) programs, inducing adhesion molecules [vascular cell adhesion molecule-1 (VCAM-1), intercellular adhesion molecule-1(ICAM-1), E-selectin], chemokines monocyte chemotactic protein-1 (MCP-1), and procoagulant mediators while suppressing eNOS activity (38, 39). Oxidative stress amplifies these pathways and can promote inflammasome activation (e.g., NLRP3), driving IL-1β/IL-18 release and endothelial injury (40). Notch1 activation and senescence-associated secretory phenotype (SASP) provide an additional feed-forward module that sustains inflammation, permeability, and NO deficiency.

Mechanistic link to endothelial phenotypes: NF-κB (p65/p50) and JAK/STAT programs translate inflammatory cues into a pro-adhesive, pro-thrombotic endothelial state by directly promoting transcription of VCAM1, ICAM1, E-selectin, MCP-1 and related chemokines, while concurrently suppressing NO signaling (via reduced Akt-eNOS activation and increased oxidative stress). Functionally, these gene programs increase leukocyte rolling/adhesion and microvascular plugging, worsen permeability and glycocalyx integrity, and contribute to impaired vasomotion—thereby connecting molecular signaling modules to clinically relevant ED phenotypes (vasomotor dysfunction, barrier disruption, and inflammatory activation) (41–43).

Definition of feed-forward loops: In this review, “feed-forward loop” refers to positive reinforcement circuits in which an initiating stressor (e.g., oxidative stress or cytokine signaling) induces mediators that further amplify the same upstream stress. For example, NF-κB can upregulate NOX components and inflammatory cytokines, increasing ROS that further activates NF-κB; similarly, Notch1/SASP-driven endothelial senescence sustains cytokine/ROS signaling and maintains chronic endothelial activation (36, 44).

EndMT cascade: persistent inflammatory/oxidative cues, disturbed flow, hypoxia, and TGF-β signaling activate small mother against decapentaplegic (SMAD) 2/3 and cooperating pathways (Notch, Wnt/β-catenin), inducing transcription factors SNAIL/SLUG, ZEB1/2, TWIST (45). This suppresses endothelial markers (VE-cadherin, CD31, eNOS) and upregulates mesenchymal programs (α-SMA, vimentin, fibronectin, collagen), promoting migration, extracellular matrix deposition, and fibrosis. EndMT therefore links chronic ED to structural vascular remodeling and organ fibrosis (Figure 2).

*In vivo* evidence for EndMT: Beyond *in vitro* marker changes, EndMT has been supported in multiple disease models using endothelial lineage tracing strategies (e.g., VE-cadherin-CreERT2 or Tie2-Cre-based reporters combined with mesenchymal marker readouts), demonstrating that a subset of fibroblast-like or smooth-muscle-like cells can derive from an endothelial lineage in settings such as cardiac, renal, and pulmonary fibrosis and advanced atherosclerotic lesions. Importantly, the magnitude of EndMT contribution appears context-dependent, and partial/“hybrid” EndMT states captured by single-cell transcriptomics suggest a spectrum rather than an all-or-none transition, which helps explain heterogeneous results across tissues and experimental systems (46–49).

#### 2.2.2 Mechanisms of reduced NO bioavailability

NO is an essential signaling molecule that plays a critical role in maintaining endothelial function and vascular homeostasis. However, various pathological conditions can lead to a decrease in NO bioavailability, which significantly contributes to ED. One of the main reasons for reduced NO availability is the impaired activity of nitric oxide synthase (NOS), especially eNOS. Several factors can hinder eNOS activity, with oxidative stress being a prominent issue in many cardiovascular diseases. For example, the buildup of ROS can cause eNOS to become uncoupled; instead of producing NO, the enzyme starts generating superoxide, which worsens oxidative stress and creates a harmful cycle of ED (50). Additionally, the presence of essential cofactors, such as tetrahydrobiopterin, is vital for eNOS to function correctly. Under oxidative stress conditions, tetrahydrobiopterin can be oxidized, leading to eNOS uncoupling and a decrease in NO production (51). Moreover, inflammatory cytokines like interleukin-6 (IL-6) can stimulate mitochondrial ROS production, which further reduces NO availability in endothelial cells (52). This intricate relationship between eNOS activity, oxidative stress, and inflammatory mediators underscores the complex regulation of NO bioavailability and its significant implications for endothelial health.

Another significant factor that contributes to the reduced bioavailability of NO is the interaction between NO and ROS, which results in the formation of peroxynitrite, a powerful oxidant that can lower NO levels. This formation occurs when superoxide reacts with NO, leading to a decrease in the availability of NO for essential processes like vasodilation and other physiological functions (53). This interaction not only diminishes NO bioavailability but also encourages protein nitration, which can negatively affect endothelial function and facilitate the progression of atherosclerosis (54). Additionally, conditions such as chronic kidney disease and diabetes mellitus have been linked to increased levels of endogenous NOS inhibitors, such as asymmetric dimethylarginine (ADMA), which further reduce NO production (55). The accumulation of these inhibitors, combined with oxidative stress, creates an environment that is harmful to endothelial function, thereby increasing cardiovascular risk.

In summary, the mechanisms that lead to reduced NO bioavailability are intricate and involve multiple factors, including impaired eNOS activity, oxidative stress, and the interplay between NO and ROS. These elements work together to cause ED, which serves as a precursor to a range of cardiovascular diseases. Gaining insight into these mechanisms is essential for creating therapeutic strategies that focus on restoring NO bioavailability and enhancing endothelial function in patients who are at risk for cardiovascular complications.

#### 2.2.3 Oxidative stress and protein tyrosine nitration

Oxidative stress, which occurs when there is an excessive buildup of ROS, plays a crucial role in causing ED, a key factor in the development of various cardiovascular diseases. This condition arises from an imbalance between the production of ROS and the body's antioxidant defense mechanisms, leading to oxidative damage to endothelial cells and impaired vascular function. Elevated levels of ROS initiate a cascade of deleterious cellular events, including lipid peroxidation, protein oxidation, and DNA damage, collectively compromising endothelial integrity and function. A specific consequence of oxidative stress is the nitration of tyrosine residues in proteins, resulting in the formation of 3-nitrotyrosine, which serves as a biomarker for nitrosative stress. This modification can alter the structure and function of proteins, thereby contributing to the advancement of vascular diseases like atherosclerosis. For example, the nitration of eNOS can hinder its activity, leading to NO bioavailability decline, which is vital for maintaining vascular tone and homeostasis. Additionally, research indicates that conditions such as diabetes and hypertension worsen oxidative stress, resulting in increased ROS production and further endothelial damage. The relationship between oxidative stress and ED underscores the importance of developing therapeutic strategies that enhance antioxidant defenses and reduce ROS accumulation to safeguard endothelial function and prevent cardiovascular complications (56–58).

Protein tyrosine nitration is an important post-translational modification that occurs during oxidative stress and is linked to the development of various diseases, particularly atherosclerosis. This modification can significantly impact protein function by changing their structure and stability, which in turn disrupts signaling pathways and cellular responses. For instance, when eNOS is nitrated, its enzymatic activity diminishes, and it becomes uncoupled, leading to reduced NO production and increased ROS generation. This process further worsens ED. Additionally, nitration affects other vital proteins that play roles in maintaining vascular health, such as calmodulin and various cytoskeletal proteins, which can hinder their normal functions and contribute to the structural and functional changes seen in atherosclerotic lesions. The detection of nitrated proteins in atherosclerotic plaques suggests that protein tyrosine nitration could be a marker for oxidative stress and vascular damage. Furthermore, the buildup of 3-nitrotyrosine in blood vessel tissues has been linked to heightened inflammation and plaque instability, both of which are critical in the progression of atherosclerosis. Gaining insight into how protein tyrosine nitration affects vascular health is essential for creating targeted therapies that aim to reduce oxidative stress and enhance endothelial function, ultimately lowering the risk of cardiovascular diseases (59–61) (Figure 1).

#### 2.2.4 Inflammation-mediated endothelial activation

Endothelial activation refers to a stimulus-induced, often reversible pro-inflammatory and pro-adhesive endothelial phenotype (e.g., TNF-α/IL-1β or LPS triggering E-selectin, VCAM-1, ICAM-1, MCP-1/IL-8, and tissue factor expression) that can precede or coexist with endothelial dysfunction. In contrast, “endothelial dysfunction” denotes a broader and usually sustained loss of homeostatic endothelial functions, including reduced NO-dependent vasodilation, impaired barrier and anticoagulant properties, and increased oxidative stress (28, 62).

Inflammation plays a crucial role in activating endothelial cells, which is essential in the development of various cardiovascular diseases. Key pro-inflammatory cytokines, including IL-6, interleukin-8 (IL-8), and monocyte chemotactic protein-1 (MCP-1), are significantly involved in this process. IL-6 promotes endothelial activation by inducing the expression of adhesion molecules such as vascular cell adhesion molecule-1 (VCAM-1) and intercellular adhesion molecule-1 (ICAM-1), which facilitate the adhesion and infiltration of leukocytes into the vascular wall. This series of events contributes to the development of atherosclerosis and other inflammatory vascular diseases (63). Similarly, IL-8, a potent chemokine, enhances the recruitment of neutrophils to inflamed tissues, further worsening ED through oxidative stress and inflammatory mediators (64). MCP-1 is vital for monocyte recruitment, promoting chronic inflammation and atherogenesis by maintaining the inflammatory environment within the vascular endothelium (65). The interactions among these cytokines not only sustain endothelial activation but also create a vicious cycle of inflammation and oxidative stress, ultimately leading to ED and an increased risk of cardiovascular events (66).

The Notch1 signaling pathway has become an important mediator of inflammation and cellular senescence in endothelial cells. It is activated in response to inflammatory stimuli and plays a vital role in maintaining the balance of endothelial function. When inflammation occurs, the activation of Notch1 can lead to the production of pro-inflammatory cytokines and adhesion molecules, which in turn promotes the activation of endothelial cells (67). Recent research has shown that exposure to particulate matter, known to induce oxidative stress and inflammation, results in increased cleavage of Notch1 and subsequent activation of its downstream signaling pathways (65). This activation not only amplifies inflammatory responses but also contributes to cellular senescence, a condition marked by irreversible cell cycle arrest and heightened secretion of pro-inflammatory factors. Endothelial cells that have entered this senescent state display altered functions, such as increased permeability and decreased nitric oxide production, which further aggravates vascular dysfunction (68). Therefore, the Notch1 signaling pathway acts as a crucial link between inflammation and ED, underscoring its potential as a therapeutic target for managing cardiovascular diseases linked to chronic inflammation (24).

In conclusion, the inflammatory mediators IL-6, IL-8, and MCP-1, as well as the Notch1 signaling pathway, are crucial in activating endothelial cells during inflammatory responses. Gaining a deeper understanding of these mechanisms offers significant insights into the pathophysiology of ED and opens up avenues for developing targeted therapeutic strategies aimed at alleviating the negative impacts of inflammation on vascular health (Figure 2).

#### 2.2.5 Endoplasmic reticulum stress and endothelial cell apoptosis

Endoplasmic reticulum (ER) stress is increasingly recognized as a significant factor contributing to ED and apoptosis, especially in cardiovascular diseases. The ER plays a vital role in protein folding, and when misfolded proteins accumulate, it triggers a cellular stress response known as the unfolded protein response. This response aims to restore normal function, but if the stress continues, it can lead to apoptosis. Key markers of ER stress, such as glucose-regulated protein 78 (GRP78) and C/EBP homologous protein (CHOP), are upregulated during these stress conditions. For example, research has demonstrated that exposure to OxLDL or high glucose levels can induce ER stress in endothelial cells, resulting in increased expression of GRP78 and CHOP, which ultimately promotes apoptosis (69, 70). The regulation of these markers is crucial, as they not only reflect the level of ER stress but also play essential roles in determining cell fate. Notably, GRP78 has a dual role; while it functions as a chaperone to help alleviate stress, prolonged activation can lead to apoptosis through pathways involving CHOP. This underscores the delicate balance that endothelial cells must maintain to survive under stress conditions.

The protein tyrosine phosphatase 1B (PTP1B) has become an important factor in the process of endothelial cell apoptosis induced by ER stress. PTP1B is recognized for its role in negatively regulating insulin receptor signaling and its involvement in the ER stress response. Research has shown that inhibiting PTP1B can reduce ER stress and enhance endothelial function. For instance, studies indicate that silencing PTP1B expression or employing specific inhibitors can prevent apoptosis in endothelial cells subjected to ER stressors such as thapsigargin or dithiothreitol (25, 71). This protective effect is linked to increased activation of eNOS and improved angiogenic capacity, highlighting the potential of targeting PTP1B as a therapeutic approach to prevent ED and apoptosis in various cardiovascular diseases. Additionally, the relationship between ER stress and apoptosis pathways, which includes the activation of caspases and the role of mitochondrial dysfunction, emphasizes the complexity of these processes in endothelial cells. Overall, gaining a deeper understanding of how ER stress and PTP1B contribute to endothelial cell apoptosis offers valuable insights into possible therapeutic targets for cardiovascular diseases associated with ED.

#### 2.2.6 Pathological significance of EndMT

EndMT is a vital biological process that significantly impacts various pathological conditions, especially in vascular remodeling and fibrosis. During EndMT, endothelial cells undergo a transformation, losing their typical characteristics and adopting mesenchymal traits, which allows them to migrate and proliferate in response to pro-inflammatory signals. This transition is particularly important in vascular diseases, as it plays a crucial role in the development of atherosclerosis and other fibrotic conditions. For example, research has demonstrated that EndMT is a key factor in the formation of fibrotic tissue in the heart, lungs, and kidneys, where endothelial cells change into myofibroblasts that produce excessive extracellular matrix components. This process not only results in tissue stiffness and impaired organ function but also worsens the pathology of chronic diseases such as diabetes and hypertension (16, 72). The significance of EndMT in vascular remodeling is highlighted by its involvement in the development of conditions like pulmonary arterial hypertension, where it contributes to the proliferation of smooth muscle cells and the narrowing of blood vessels, ultimately leading to increased vascular resistance and right heart failure (15, 73). The relationship between EndMT and inflammation is significant, as inflammatory cytokines like transforming growth factor-beta (TGF-β) and interleukin-1beta (interleukin-1β) can trigger this transition in endothelial cells. This process often leads to increased vascular permeability and the infiltration of leukocytes, which further fuels the inflammatory cycle and contributes to tissue damage (74, 75). The interaction between EndMT and inflammatory processes underscores the potential for therapeutic targeting of this pathway. For instance, strategies aimed at inhibiting TGF-beta signaling have been suggested to prevent EndMT and its related fibrotic outcomes, indicating that modifying this transition could pave the way for new treatments for chronic inflammatory and fibrotic diseases (15, 76).

EndMT plays a significant role not only in fibrosis but also in cancer biology, where it aids in tumor progression and metastasis. Tumor cells can harness the mechanisms of EndMT to acquire migratory and invasive characteristics, which enable them to spread to distant locations (77, 78). This highlights the dual nature of EndMT, functioning as a physiological process vital for tissue repair while also serving as a pathological mechanism that can result in negative outcomes in various disease contexts. Consequently, it is crucial to comprehend the molecular pathways that regulate EndMT to develop targeted therapies aimed at alleviating its detrimental effects while maintaining its beneficial roles in tissue homeostasis and repair (76, 79).

In summary, EndMT is a crucial pathological mechanism that has extensive implications for vascular remodeling, fibrosis, inflammation, and cancer. The regulation of EndMT is intricate and affected by multiple factors, such as inflammatory cytokines and mechanical stress. By targeting EndMT, there is potential for developing innovative therapeutic strategies to tackle various diseases associated with vascular dysfunction and fibrosis, which could enhance patient outcomes in conditions like atherosclerosis, pulmonary hypertension, and fibrotic disorders (15, 80) (Figure 2).

### 2.3 The association between ED and metabolic diseases

#### 2.3.1 Mechanisms of ED in diabetes

Dominant ED phenotypes in diabetes include impaired endothelium-dependent vasodilation (NO deficiency), inflammatory endothelial activation, and progressive barrier dysfunction that can be reinforced by EndMT in chronic vasculopathy. Mechanistically, hyperglycemia/insulin resistance converge on PI3K/Akt-eNOS suppression, ADMA/arginase-driven substrate limitation, BH4 oxidation and eNOS uncoupling, and NOX/mitochondrial ROS amplification; these signals engage NF-κB/JAK/STAT programs to increase VCAM-1/ICAM-1 and cytokine production, thereby linking metabolic stress to vascular inflammation.

ED is a crucial aspect of diabetes mellitus, especially associated with insulin resistance, which significantly impacts vascular health. Insulin resistance, defined as the reduced effectiveness of insulin in performing its biological roles, disrupts endothelial function through various mechanisms. A key pathway involved is the impairment of insulin signaling, particularly affecting the PI3K/Akt pathway, vital for activating eNOS. In individuals with diabetes, the activation of this pathway is often diminished, leading to decreased production of NO, an essential mediator for vasodilation and maintaining endothelial health (81). Additionally, the buildup of advanced glycation end-products (AGEs) in diabetes worsens oxidative stress, resulting in increased generation of ROS that further compromise endothelial function by reducing NO availability and fostering inflammation (82). This ED arises not only from hyperglycemia but also from a complex interaction of metabolic disturbances, inflammatory responses, and oxidative stress, all of which contribute to the advancement of vascular complications in diabetes (83). Addressing these pathways, especially through pharmacological agents that improve insulin sensitivity or directly activate the PI3 K/Akt/eNOS pathway, offers a promising therapeutic approach to alleviate ED in diabetic patients.

The role of macrophages in the development of ED in diabetes is becoming increasingly important, especially when considering macrophage polarization. In the context of diabetes, there is a notable shift in macrophage polarization from the anti-inflammatory M2 phenotype to the pro-inflammatory M1 phenotype, which plays a significant role in causing damage to the endothelium (84). M1 macrophages produce a wide range of inflammatory cytokines and chemokines that worsen ED, resulting in increased vascular permeability, enhanced adhesion of leukocytes, and the promotion of atherogenesis. This shift in polarization is influenced by several factors, particularly hyperglycemia, which encourages the release of pro-inflammatory substances and heightens oxidative stress within the blood vessels (85). When M1 macrophages become activated, they interact with endothelial cells, leading to the increased expression of adhesion molecules like ICAM-1 and VCAM-1. This process facilitates the infiltration of more inflammatory cells, creating a vicious cycle of endothelial damage. Additionally, the inflammatory cytokines secreted by M1 macrophages can hinder the ability of endothelial cells to regenerate, resulting in a reduced capacity for angiogenesis and contributing to the onset of diabetic complications such as retinopathy and nephropathy (86). Therefore, therapeutic strategies that focus on modifying macrophage polarization either by encouraging M2 polarization or by inhibiting M1 activation could offer a promising new approach to alleviate ED and its related complications in diabetes (Table 2).

**Table 2:** Candidate biomarkers of endothelial dysfunction: what they reflect, representative disease contexts, and translational considerations.

Biomarker/readout	What it reflects	Representative disease contexts	Therapy/translation notes	Key references
NO metabolites; eNOS activity indices; ADMA	NO bioavailability and eNOS coupling; substrate competition	Diabetes, obesity, INOCA, hypertension	Responsive to lifestyle and cardiometabolic therapies; useful for mechanistic monitoring of the NO-ROS axis	(50–55, 113)
3-nitrotyrosine; oxidized LDL; oxidative stress panels	Nitrosative/oxidative stress and ONOO− formation	Atherosclerosis, diabetes, OSA	Tracks antioxidant/anti-inflammatory strategies; interpret with context due to systemic sources	(56–61, 112)
Soluble ICAM-1/VCAM-1; E-selectin; MCP-1	Inflammatory endothelial activation and leukocyte recruitment	CAD/atherosclerosis, metabolic syndrome, OSA	Potential surrogate markers for anti-inflammatory therapies; linked to vascular inflammation	(62–66, 111)
Circulating endothelial cells (CECs)	Endothelial injury and shedding	Stroke, diabetes microangiopathy, systemic inflammation	Promising for dynamic monitoring; requires standardization and reference ranges	(104–106)
Endothelial EVs/microparticles	Activation/apoptosis and barrier perturbation signatures	Diabetes, stroke, inflammatory states	High potential but sensitive to pre-analytical variables	(109)
miRNAs/lncRNAs (endothelial-enriched panels)	Regulatory programs of endothelial stress responses	INOCA, diabetes, atherosclerosis (context-dependent)	High discovery potential; needs validated panels and reproducible assays	(133–135, 141, 142)
Flow-mediated dilation (FMD); PAT/RHI	Macrovascular endothelial-dependent vasodilation	Cardiometabolic risk states; CAD risk prediction	Non-invasive and scalable; influenced by operator/physiologic confounders	(100, 101, 120, 121)
Coronary function testing (acetylcholine, CFR/IMR); PET/CMR perfusion	Coronary microvascular and epicardial vasomotor function	INOCA, microvascular angina	Mechanistically aligned endpoints; availability and invasiveness vary	(102, 103)
BBB permeability imaging and neurovascular coupling metrics	Barrier integrity and microvascular inflammation	Acute ischemic stroke	Useful for prognosis and monitoring; needs standardized protocols	(104–106)

Key references correspond to the numbered reference list in the manuscript.

#### 2.3.2 The impact of obesity and adipokines on endothelial function

In obesity, ED is often dominated by low-grade inflammatory activation and reduced NO bioavailability, with additional contributions from oxidative stress and perivascular adipose tissue dysfunction. A practical cascade begins with adipokine imbalance (increased leptin/resistin and reduced adiponectin) together with elevated free fatty acids, which activate TLR/NF-κB signaling and promote NOX-derived ROS production; these changes inhibit and/or uncouple eNOS and impair vasodilation, while also increasing endothelial permeability and leukocyte recruitment.

The relationship between obesity and ED is well-established, with adipokines playing a crucial role in this connection. Obesity is marked by an excessive buildup of adipose tissue, which alters the secretion of adipokines and results in a state of chronic low-grade inflammation. Among these adipokines, leptin has received considerable attention due to its dual role in regulating energy and vascular function. Produced primarily by adipocytes, leptin signals the hypothalamus about the body's energy status; however, its elevated levels in obesity are linked to ED. Research indicates that leptin can trigger oxidative stress and inflammation in endothelial cells, which impairs vasodilation and increases vascular resistance. This dysregulation is especially evident in metabolic syndrome, where the interaction between high leptin levels and other pro-inflammatory cytokines worsens ED, leading to cardiovascular complications (87, 88).

Emerging evidence suggests that the effects of leptin on endothelial function may differ between sexes, with distinct mechanisms at play in males and females. In males, the activation of leptin receptors in endothelial cells seems to provide a protective effect against ED by enhancing the production of NO and promoting vasodilation. In contrast, females may experience a different response to leptin due to the influence of sex hormones, such as estrogen, which can modify leptin signaling pathways. This variation in response is especially significant during pregnancy, where elevated leptin levels are noted in conditions like gestational hypertension and preeclampsia. In these situations, leptin contributes to systemic ED, leading to increased vascular resistance and impaired blood flow, which are critical factors in the development of pregnancy-related hypertensive disorders. Understanding these gender-specific mechanisms is essential for creating targeted therapeutic strategies that take into account the unique physiological responses to leptin in different populations (89, 90).

Leptin plays a crucial role in gestational hypertension and preeclampsia, highlighting its importance in pregnancy-related ED. In cases of preeclampsia, the placenta produces excessive amounts of leptin, which is linked to increased systemic vascular resistance and activation of the endothelium. These elevated leptin levels contribute to the inflammatory environment seen in preeclampsia, potentially enhancing the production of other pro-inflammatory cytokines and worsening ED. This series of events leads to the typical clinical signs of preeclampsia, such as hypertension and proteinuria. Additionally, the connection between high leptin levels and negative fetal outcomes emphasizes the need to understand how leptin affects both maternal and fetal health. Research suggests that interventions aimed at adjusting leptin levels or its signaling pathways could provide promising therapeutic options for managing preeclampsia and improving outcomes for both mothers and their babies. Therefore, the complex relationship between obesity, leptin, and endothelial function during pregnancy calls for further exploration to identify new prevention and treatment strategies (90, 91).

#### 2.3.3 Endothelial injury in diabetes-related microvascular complications

In diabetic microvascular disease, the dominant phenotype is microvascular barrier injury (capillary leakage, glycocalyx loss) and rarefaction rather than isolated vasomotor dysfunction. Key nodes include ROS-driven junctional disruption, Ras homolog gene family member A/Rho-associated coiled-coil forming protein kinase (RhoA/ROCK)-mediated cytoskeletal contraction, inflammatory cytokines, and, in some contexts, EndMT programs that promote perivascular fibrosis and capillary dropout.

The apelin/apelin receptor (APJ) signaling pathway has emerged as a crucial factor in ED, particularly concerning microvascular complications related to diabetes. Apelin, an endogenous peptide, and its receptor APJ play significant roles in various cardiovascular processes, such as angiogenesis, vascular permeability, and maintaining endothelial integrity. In diabetic conditions, which are marked by chronic hyperglycemia and oxidative stress, endothelial cell functionality is severely impaired, resulting in increased vascular permeability and subsequent microvascular complications. Research indicates that administering apelin can counteract the harmful effects of elevated glucose levels on endothelial cells by enhancing NO production, promoting cell survival, and reducing apoptosis. Specifically, apelin has been found to improve endothelial function by activating the APJ receptor, which in turn triggers downstream signaling pathways, including the PI3K/Akt pathway. This activation increases the expression and activity of eNOS, thereby enhancing NO availability and promoting vasodilation. Additionally, apelin has demonstrated the ability to reduce inflammation and oxidative stress, both of which are significant contributors to ED in diabetes. By restoring endothelial function through the apelin/APJ signaling axis, therapeutic strategies that target this pathway may provide promising options for preventing and treating microvascular complications associated with diabetes, ultimately leading to improved patient outcomes in diabetic populations (92, 93).

Microvascular permeability plays a crucial role in the development of diabetic cardiomyopathy, which is marked by structural and functional heart abnormalities that occur without the presence of coronary artery disease. In individuals with diabetes, the heightened permeability of microvessels results in the leakage of plasma proteins and fluids into the surrounding tissue, leading to edema and compromised heart function. For example, in diabetic cardiomyopathy and heart failure with preserved ejection fraction, coronary microvascular endothelial activation/dysfunction reduces NO/cyclic guanosine monophosphate (cGMP) signaling, promotes capillary leak and leukocyte/macrophage infiltration, and triggers TGF-β–driven fibroblast activation, thereby accelerating interstitial fibrosis, concentric remodeling, and progressive myocardial stiffening (94–98) Additionally, elevated levels of AGEs and ROS in diabetes worsen endothelial damage, further increasing vascular permeability. This series of events can ultimately lead to myocardial ischemia, heart muscle thickening, and progressive heart failure. Research has shown that interventions designed to restore endothelial function and decrease microvascular permeability can slow the progression of diabetic cardiomyopathy. For example, certain medications that boost NO availability or target inflammatory processes have demonstrated potential in reducing the negative impacts of increased microvascular permeability on heart function. Therefore, comprehending the link between microvascular permeability and cardiomyopathy is vital for creating effective treatment approaches to address the cardiovascular issues that arise from diabetes (93, 99).

### 2.4 Clinical manifestations and risk prediction of ED in cardiovascular diseases

#### 2.4.1 Coronary artery disease (CAD) and ischemia with non-obstructive coronary arteries (INOCA)

In CAD and INOCA, ED is frequently expressed as a vasomotor phenotype (impaired NO-mediated dilation, microvascular dysfunction, and/or spasm) together with inflammatory endothelial activation that accelerates plaque progression. Mechanistically, the NO-ROS vicious cycle: NOX- and mitochondrial ROS production and eNOS uncoupling mutually reinforce each other, which integrates with NF-κB-driven adhesion molecule expression and, in chronic disease, fibrotic remodeling modules (including EndMT-like signatures in plaque and perivascular compartments).

Flow-mediated dilation (FMD) is an important non-invasive technique used to evaluate endothelial function, especially relevant in cases of CAD and INOCA. ED has emerged as a crucial factor contributing to cardiovascular issues and increased mortality, particularly among individuals who experience angina but do not show obstructive lesions on coronary angiography. FMD assesses how well blood vessels can expand in response to heightened blood flow, serving as an indicator of endothelial health. Research indicates that reduced FMD is linked to negative cardiovascular events, such as myocardial infarction and heart failure, highlighting its significance in predicting patient outcomes. In individuals with INOCA, ED can result in microvascular ischemia, which may cause angina symptoms even in the absence of obstructive coronary lesions. Moreover, FMD has been associated with systemic conditions like hypertension and diabetes, which can further impair endothelial function. The clinical relevance of FMD in patients with INOCA lies in its ability to inform treatment strategies aimed at enhancing endothelial health and improving overall patient outcomes. For example, lifestyle changes and medications designed to support endothelial function can be customized based on FMD findings, allowing for a more personalized management approach for INOCA. Additionally, ongoing studies are investigating the connections between FMD and various biomarkers of ED, which could improve the predictive power of FMD in clinical practice. Therefore, FMD not only acts as a diagnostic measure but also represents a potential target for therapy, underscoring the importance of integrating it into standard clinical care for patients suspected of having INOCA (100, 101).

The intricate relationship between ED, coronary vasospasm, and myocardial ischemia is particularly evident in patients diagnosed with INOCA. ED refers to the endothelium's diminished ability to regulate vascular tone and maintain homeostasis, which can result in impaired vasodilation and an increased likelihood of vasospasm. In patients with INOCA, coronary vasospasm can occur even when there are no significant obstructive lesions, often presenting as episodes of angina or myocardial ischemia. This situation underscores the necessity of understanding the mechanisms behind ED and its role in triggering vasospastic events. Research has shown that factors such as oxidative stress, inflammation, and autonomic dysregulation play a role in the pathophysiology of ED, which in turn promotes vasospasm. Additionally, the interaction among these mechanisms can worsen myocardial ischemia, potentially leading to adverse cardiovascular events. Clinical evaluations, including provocative testing with agents like acetylcholine, can assist in identifying patients with vasospastic angina, offering valuable insights into the causes of their symptoms. Effective management strategies for INOCA typically involve addressing both ED and vasospasm through lifestyle changes, pharmacotherapy, and, in some cases, invasive procedures. Understanding the relationship between these factors is essential for optimizing treatment and enhancing outcomes for patients with INOCA, thereby addressing a significant gap in current cardiovascular care (102, 103).

#### 2.4.2 ED in acute ischemic stroke

In acute ischemic stroke, ED is dominated by barrier failure at the blood-brain barrier (BBB) and microvascular inflammation, which drive edema, hemorrhagic transformation risk, and impaired reperfusion. A mechanistic cascade is triggered by ischemia/reperfusion, whereby ROS generation and damage-associated molecular pattern (DAMP)/TLR signaling drive NF-κB activation and matrix metalloproteinases (MMP) upregulation, leading to tight-junction disruption and glycocalyx shedding and, subsequently, enhanced leukocyte adhesion and microthrombosis.

ED is a critical factor in the pathophysiology of acute ischemic stroke, significantly affecting both the occurrence of complications and the overall prognosis for those affected. Research has established a strong connection between ED and negative outcomes following a stroke, including the formation of parenchymal hematomas, which can arise after thrombectomy procedures. For example, patients showing signs of ED are at a heightened risk for such complications, resulting in worse clinical outcomes and increased mortality rates (104). The mechanisms behind this involve a series of inflammatory responses and compromised vascular integrity, which can worsen ischemic injury and impede recovery. Additionally, ED is linked to various comorbidities, such as hypertension and diabetes, which are common among stroke patients and can intensify the severity of the ischemic event. This complex relationship highlights the significance of evaluating endothelial function as a potential prognostic marker in individuals experiencing acute ischemic stroke. New evidence indicates that therapeutic approaches aimed at enhancing endothelial function may help reduce some complications related to stroke and improve recovery outcomes. For instance, the use of angiotensin-converting enzyme inhibitors has been investigated for their protective effects on the endothelium, potentially leading to better clinical results for stroke patients (105). Therefore, understanding and addressing ED is essential for optimizing management strategies in acute ischemic stroke.

Recent advancements in our understanding of endothelial progenitor cells (EPCs) and their therapeutic potential have opened new avenues for treating ED in acute ischemic stroke. EPCs play a vital role in vascular repair and regeneration, yet their mobilization and function are often compromised in patients with cardiovascular diseases, including stroke. Research suggests that enhancing the availability and functionality of EPCs could significantly improve outcomes in acute ischemic stroke by promoting endothelial repair and restoring vascular integrity (104). Various pharmacological agents are currently being investigated for their ability to mobilize EPCs or enhance their function. For example, therapies targeting the sphingosine-1-phosphate receptor have shown promise in preclinical models, indicating that modulating this pathway could lead to improved endothelial health and function after a stroke (106). Moreover, the application of stem cell therapy, particularly using EPCs derived from bone marrow or peripheral blood, is being explored as a potential treatment for restoring endothelial function and promoting neuroprotection following ischemic events. Early clinical trials have demonstrated that administering EPCs can lead to better neurological outcomes and reduced infarct size, underscoring their therapeutic potential (105). However, challenges persist in optimizing the timing, dosage, and delivery methods of these therapies to maximize their efficacy in clinical settings. Ongoing research aims to clarify the precise mechanisms through which EPCs exert their beneficial effects and to develop strategies for effectively integrating these therapies into standard stroke management protocols. The promising results from these studies highlight the need for continued exploration of EPCs and pharmacological interventions aimed at alleviating ED in patients with acute ischemic stroke, ultimately striving to enhance recovery.

#### 2.4.3 Endothelial injury and myocardial injury in patients with obstructive sleep apnea

In obstructive sleep apnea, intermittent hypoxia produces a mixed ED phenotype characterized by impaired vasodilation and barrier dysfunction driven by oxidative stress and sympathetic/inflammatory activation. Intermittent hypoxia increases mitochondrial- and NOX-derived ROS, which reduces NO bioavailability and promotes ONOO^−^ formation, while concomitantly activating NF-κB and inducing adhesion molecule expression; collectively, these changes are consistent with microvascular injury and an increased cardiovascular risk.

Obstructive sleep apnea (OSA) is increasingly recognized as a significant contributor to cardiovascular issues, particularly through mechanisms involving ED and myocardial injury. Research has focused on the relationship between peripheral arterial tension measurements and high-sensitivity cardiac troponin I (hs-cTnI) levels to better understand the cardiovascular implications of OSA. Studies indicate that patients with moderate to severe OSA show elevated levels of hs-cTnI, which signals myocardial injury and correlates with impaired peripheral arterial function. For instance, a recent cross-sectional study involving hypertensive patients found that those with OSA had significantly higher hs-cTnI levels compared to those without OSA, suggesting that the intermittent hypoxia characteristic of OSA worsens myocardial injury (107). Additionally, peripheral arterial tension measurements, such as FMD, are utilized to evaluate endothelial function, with lower FMD values indicating ED. This dysfunction is frequently observed in OSA patients, as the repeated cycles of hypoxia and reoxygenation lead to oxidative stress and inflammation, further compromising endothelial function and increasing cardiovascular risk (26). The connection between peripheral arterial tension and hs-cTnI levels highlights the importance of monitoring these parameters in patients with OSA, as they may serve as valuable indicators of cardiovascular health and inform therapeutic interventions.

ED has become a significant predictor of subclinical myocardial injury in patients with OSA. The mechanisms behind this relationship involve a combination of intermittent hypoxia, oxidative stress, and inflammation, all of which lead to injury of the endothelial cells and subsequent damage to the heart muscle. In a cohort study of male patients diagnosed with OSA, nearly half showed signs of ED, which was significantly linked to detectable levels of hs-cTnI, a biomarker that indicates myocardial injury (108). The study found that patients with ED had a higher rate of detectable hs-cTnI levels compared to those without dysfunction, suggesting that impaired endothelial function may occur before and predict myocardial injury in these patients. Additionally, the research indicated that factors such as the apnea-hypopnea index and oxygen desaturation index were related to the severity of ED, highlighting that the extent of hypoxia experienced during sleep directly affects cardiovascular health (27). These findings emphasize the need for early detection and management of ED in patients with OSA, as addressing this issue could reduce the risk of developing more serious cardiovascular problems, including heart failure and ischemic events. Future studies should aim to clarify how ED leads to myocardial injury and investigate targeted treatments to enhance endothelial function in individuals with OSA.

### 2.5 Research progress on biomarkers of ED

To improve readability and clinical utility, we summarize biomarkers of endothelial dysfunction (ED) in a phenotype- and module-oriented manner (Table 2). Specifically, we group markers that reflect endothelial activation and inflammation, NO-ROS imbalance (including eNOS uncoupling and oxidative/nitrative stress), barrier vulnerability, and remodeling programs such as EndMT. We also align circulating biomarkers with representative functional and imaging readouts and highlight typical disease contexts and translational considerations to facilitate mechanism-guided interpretation in clinical practice.

#### 2.5.1 Traditional biomarkers

ED plays a crucial role in the development of various cardiovascular diseases, and identifying traditional biomarkers has been essential in understanding its significance. Among these biomarkers, endothelial cell adhesion molecules, especially E-selectin and CD62E+ microparticles, have become important indicators of endothelial activation and dysfunction. E-selectin is a cell adhesion molecule found on activated endothelial cells, which aids in the adhesion of leukocytes to the endothelium—a vital step in the processes of inflammation and atherosclerosis. Increased levels of E-selectin in the bloodstream have been linked to several cardiovascular conditions, suggesting ongoing endothelial activation and inflammation (62). Furthermore, CD62E+ microparticles, which are released from activated endothelial cells, act as markers of endothelial injury and dysfunction. Their presence in the circulation is associated with negative cardiovascular events, underscoring their potential usefulness in clinical practice for risk assessment and monitoring disease progression (109). These biomarkers not only indicate the condition of the endothelium but also shed light on the underlying mechanisms involved in vascular pathology.

Inflammatory factors such as C-reactive protein (CRP) and IL-6 play a significant role in ED. CRP is a well-known marker of systemic inflammation that has been associated with ED and an increased risk of cardiovascular disease. When CRP levels are elevated, it indicates an inflammatory state that can worsen endothelial injury and promote the development of atherosclerosis (110). Similarly, IL-6, which is a pro-inflammatory cytokine, contributes to the inflammatory response and has been found to correlate with ED across various populations, including individuals with chronic kidney disease and diabetes (111). The relationship between these inflammatory markers and endothelial function highlights the necessity of a comprehensive approach to evaluating cardiovascular risk, as it involves both inflammatory and endothelial aspects of the disease.

OxLDL and asymmetric dimethylarginine (ADMA) are two traditional biomarkers that have gained significant attention in the study of ED. OxLDL is a modified form of low-density lipoprotein that plays a detrimental role in endothelial health by promoting oxidative stress and inflammation, which in turn contributes to the development of atherosclerosis (112). The presence of OxLDL in the bloodstream serves as an indicator of a negative lipid profile and an increased risk of cardiovascular diseases. Conversely, ADMA acts as an endogenous inhibitor of nitric oxide synthase, resulting in decreased availability of nitric oxide and compromised endothelial function. Elevated ADMA levels have been linked to several cardiovascular issues, such as hypertension and coronary artery disease, highlighting its importance as a biomarker for evaluating endothelial health (113). Measuring these biomarkers can offer valuable insights into the underlying mechanisms of ED and inform therapeutic strategies aimed at restoring endothelial integrity.

In summary, traditional biomarkers like E-selectin, CD62E+ microparticles, CRP, IL-6, OxLDL, and ADMA are crucial for evaluating ED. Measuring these biomarkers not only helps in diagnosing and stratifying the risk of cardiovascular diseases but also deepens our understanding of the underlying pathophysiological mechanisms. As research continues to advance, incorporating these biomarkers into clinical practice could enhance patient outcomes by enabling early detection and targeted treatment strategies for ED and its related complications.

#### 2.5.2 Emerging biomarkers

Matrix metalloproteinases (MMPs), especially MMP-7 and MMP-9, have become important biomarkers for ED. MMPs are a group of zinc-dependent endopeptidases that are essential for breaking down components of the extracellular matrix, which is crucial for maintaining the integrity of blood vessels. Increased levels of MMP-7 and MMP-9 have been linked to various cardiovascular diseases, indicating their roles in endothelial injury and the remodeling processes that follow. For example, research has shown that MMP-9 levels are associated with the severity of ED, as it is released from activated endothelial cells and infiltrating leukocytes during inflammation. In contrast, MMP-7 is involved in regulating the proliferation and migration of vascular smooth muscle cells, which contributes to neointimal hyperplasia after vascular injury. The clinical significance of these biomarkers is highlighted by their potential to act as prognostic indicators for cardiovascular events; higher levels of MMP-7 and MMP-9 have been associated with worse outcomes in patients suffering from atherosclerosis and coronary artery disease. Additionally, targeting these MMPs in treatment may provide new strategies to alleviate ED and its consequences in cardiovascular diseases (62, 89).

Angiopoietin-like protein 2 (ANGPTL2), endoglin, and Annexin V+ apoptotic microparticles represent a new frontier in identifying biomarkers for ED. ANGPTL2 is a secreted protein that promotes inflammation and increases endothelial permeability, contributing to vascular dysfunction. Elevated levels of ANGPTL2 have been linked to various cardiovascular diseases, highlighting its potential as a biomarker for endothelial injury. Similarly, endoglin, which acts as a co-receptor for TGF- beta, serves as another promising biomarker reflecting endothelial activation and dysfunction. Increased endoglin levels indicate vascular remodeling and are associated with adverse cardiovascular outcomes. Furthermore, Annexin V+ apoptotic microparticles, released from dying endothelial cells, are connected to heightened vascular permeability and inflammation. These microparticles can indicate endothelial cell apoptosis and dysfunction. Together, these biomarkers enhance our understanding of the pathophysiological mechanisms behind ED and may aid in the early diagnosis and therapeutic monitoring of patients with cardiovascular diseases (62, 89).

The monocyte-to-high-density lipoprotein cholesterol ratio (MHR) has emerged as a promising new biomarker for evaluating ED, especially in individuals with type 2 diabetes. An elevated MHR indicates a pro-inflammatory state, characterized by an increase in monocytes, which are known contributors to vascular inflammation and the development of atherosclerosis. Recent research has shown a significant link between high MHR levels and impaired endothelial function, as assessed through flow-mediated dilation. This relationship implies that MHR could be a straightforward and cost-effective marker for identifying ED in clinical settings. Additionally, MHR has been found to predict cardiovascular events across various populations, including those with diabetes and metabolic syndrome. The strength of MHR as a biomarker lies in its capacity to combine both inflammatory and lipid profiles, offering a comprehensive perspective on cardiovascular risk. Consequently, monitoring MHR could improve risk assessment and inform therapeutic strategies aimed at enhancing endothelial function while reducing cardiovascular morbidity and mortality (23, 114).

#### 2.5.3 Limitations and future directions of clinical applications of biomarkers

The clinical application of biomarkers in medical practice holds significant promise; however, notable limitations continue to impede their widespread use and effectiveness. A major challenge is the insufficient sensitivity and specificity of current biomarkers, which can result in misdiagnosis or delays in treatment. For example, in the case of sepsis, no single biomarker has proven adequate to definitively confirm or exclude the condition, highlighting the need for a multi-biomarker strategy to improve diagnostic accuracy (115). Additionally, the reproducibility of biomarker studies is a pressing issue, as many proposed biomarkers do not yield consistent results across various populations and study designs. This inconsistency can stem from factors such as variations in sample handling, assay techniques, and patient demographics, complicating the validation process and restricting the generalizability of the findings (116).

The integration of biomarkers into clinical practice faces challenges due to the complexity of biological systems. Traditional biomarkers often fail to capture the intricate network of molecular interactions that define various disease states. This limitation has prompted the development of network biomarkers, which take into account the relationships among multiple biomolecules, potentially offering a more stable and reliable framework for diagnosis (117). However, moving from traditional biomarkers to network biomarkers requires additional research to clarify their clinical utility and to establish standardized methodologies for their evaluation.

Another significant limitation is the dynamic nature of disease progression, which traditional static biomarkers fail to capture effectively. The emergence of dynamic network biomarkers offers a promising avenue, as they have the potential to identify pre-disease states and monitor disease progression in real-time, thus providing a more proactive approach to patient management (117). However, the clinical implementation of dynamic network biomarkers is still in its early stages, necessitating extensive validation and refinement before they can be routinely integrated into clinical practice.

Moreover, the regulatory landscape surrounding biomarker development presents further challenges. The approval process for new biomarkers is often lengthy and complex, typically necessitating substantial evidence of clinical benefit before they can be integrated into clinical practice. This regulatory burden can hinder innovation and postpone the availability of potentially life-saving diagnostic tools (118).

Looking ahead, future research should prioritize overcoming these limitations by fostering collaborative efforts among multidisciplinary teams that include clinicians, researchers, and regulatory bodies. Establishing standardized protocols for the discovery and validation of biomarkers is crucial for enhancing both reproducibility and reliability. Furthermore, utilizing advancements in technology, such as machine learning and artificial intelligence, could aid in the identification of novel biomarkers and enhance the interpretation of complex biological data (119).

In conclusion, biomarkers present considerable promise for improving clinical practice; however, their existing limitations require a dedicated effort to enhance their application and validation. By adopting innovative strategies and encouraging collaboration across various disciplines, the field of biomarker research can progress toward more effective diagnostic and therapeutic methods, ultimately leading to better patient outcomes.

### 2.6 Diagnosis methods and technological advances in ED

Diagnosis of ED benefits from a tiered strategy that combines functional testing, imaging-based phenotyping, and molecular profiling. Functional assays directly quantify NO-dependent vasoreactivity and microvascular responses, whereas imaging can capture vascular inflammation, remodeling, and tissue-level consequences. Molecular approaches, including transcriptomic/proteomic and epigenetic profiling, provide mechanistic resolution and may nominate biomarkers and targets. Integrating these modalities improves phenotype assignment and supports longitudinal monitoring in clinical and research settings.

#### 2.6.1 Functional testing techniques

FMD is an important non-invasive technique that assesses endothelial function by measuring how blood vessels respond to increased blood flow. This method is based on the principle that endothelial cells release NO, a strong vasodilator, in response to shear stress caused by heightened blood flow. The FMD test usually involves temporarily blocking a peripheral artery, commonly the brachial artery, with a cuff, and then releasing the cuff to induce hyperemia. The resulting increase in the diameter of the blood vessel is measured using high-resolution ultrasound. Research has shown that FMD is a reliable indicator of endothelial health; impaired FMD is linked to various cardiovascular risk factors such as hypertension, diabetes, and hyperlipidemia (120). Additionally, FMD has prognostic significance, as diminished endothelial function is associated with a higher risk of cardiovascular events and mortality (68). However, it is important to recognize that several factors, including age, sex, and existing comorbidities, can influence FMD results, potentially affecting its reliability as a standalone diagnostic tool. Recent technological advancements have enhanced the accuracy of FMD measurements, making it a valuable resource in both clinical and research environments for evaluating endothelial function and assessing the effectiveness of therapeutic interventions aimed at improving vascular health (121).

The assessment of endothelial-dependent blood flow responses and pulse wave velocity (PWV) offers essential insights into vascular health and endothelial function. Endothelial-dependent blood flow response is typically evaluated using techniques like reactive hyperemia, which measures the increase in blood flow following a brief period of occlusion. This response is mainly driven by the endothelium's ability to release vasodilators such as nitric oxide in reaction to shear stress (122). In clinical practice, these measurements can help detect early signs of ED, a precursor to atherosclerosis and other cardiovascular diseases. Conversely, pulse wave velocity serves as a measure of arterial stiffness, determined by the speed at which pressure waves travel through the arteries. An increased PWV indicates arterial stiffness, which is frequently linked to ED and heightened cardiovascular risk (123). Research has demonstrated that endothelial-dependent blood flow responses and PWV are interconnected; impaired endothelial function can lead to increased arterial stiffness, thereby worsening cardiovascular risk (124). By integrating these assessments, we can deepen our understanding of the mechanisms underlying vascular diseases and assist in developing targeted therapeutic strategies aimed at improving endothelial function and overall cardiovascular health. Furthermore, employing these techniques in longitudinal studies can yield valuable data on the progression of ED and its association with various cardiovascular risk factors over time (125).

#### 2.6.2 Imaging techniques

Coronary computed tomography angiography (CTA) has become an essential non-invasive imaging technique for evaluating coronary artery disease, especially regarding ED. A notable advancement in this area is the assessment of pericardial adipose tissue attenuation (PCAT) values, which act as a surrogate marker for coronary inflammation and endothelial health. Recent studies indicate that higher PCAT attenuation values are associated with the presence of coronary artery disease and reflect underlying ED, making it a significant tool for risk stratification concerning cardiovascular events (120). The biological basis for this relationship stems from the close proximity of pericardial adipose tissue to the coronary arteries, where it can affect vascular function by secreting pro-inflammatory cytokines and adipokines. In patients with coronary artery disease, changes in PCAT attenuation have been associated with heightened inflammatory activity, a key feature of ED. Moreover, combining PCAT attenuation values with traditional coronary CTA findings improves diagnostic accuracy in identifying patients at greater risk for adverse cardiovascular outcomes. This integrated approach not only supports the early detection of ED but also enables personalized treatment strategies aimed at enhancing vascular health. As the field advances, additional research is needed to standardize PCAT measurement protocols and confirm their clinical relevance across various populations, particularly among those with metabolic syndrome and other cardiovascular disease risk factors (126).

The evolution of non-invasive vascular function testing devices has significantly advanced the assessment of endothelial function, offering clinicians essential insights into cardiovascular health without the need for invasive procedures. Techniques such as FMD, laser speckle contrast imaging, and near-infrared spectroscopy have become reliable methods for evaluating endothelial responsiveness and microvascular function (127). FMD, often considered the gold standard, measures the change in diameter of a blood vessel in response to increased blood flow, which reflects the endothelium's ability to produce nitric oxide, a key mediator of vasodilation. This method has been extensively validated and is widely utilized in both clinical and research settings to assess ED across various populations, including individuals with diabetes, hypertension, and chronic kidney diseas (128). Furthermore, emerging technologies like optical coherence tomography and magnetic resonance imaging are being investigated for their potential to visualize endothelial function at the microvascular level, thereby providing a more comprehensive understanding of vascular health. The integration of these non-invasive techniques into routine clinical practice not only enhances the early detection of ED but also facilitates the monitoring of therapeutic interventions over time. As research continues to clarify the mechanisms underlying ED, the development and refinement of these non-invasive tools will be vital in advancing personalized medicine approaches and improving cardiovascular outcomes (129).

#### 2.6.3 Molecular biology and gene expression analysis

ED plays a crucial role in the development of various cardiovascular diseases, making it essential to understand the underlying molecular biology for the creation of targeted therapies. Recent research has emphasized the significance of specific gene and protein expression profiles in endothelial cells, shedding light on their contributions to vascular health and disease. For example, eNOS is vital for maintaining vascular homeostasis by regulating the production of NO, a key mediator of vasodilation. When eNOS expression is dysregulated, it can lead to reduced NO bioavailability, which contributes to ED and the advancement of atherosclerosis (110). Additionally, advancements in liquid biopsy technologies and molecular biology techniques have facilitated the discovery of new biomarkers linked to ED, such as endoglin and endocan, which have been associated with vascular injury and inflammation (130). The combination of transcriptomic and proteomic data has offered a detailed perspective on the specific expression landscape of endothelial cells, indicating that changes in gene expression can significantly affect endothelial cell function and play a role in the onset of cardiovascular diseases (131). Furthermore, the influence of epigenetic modifications, including DNA methylation and histone modifications, on endothelial gene expression has garnered attention, suggesting that these mechanisms could be potential therapeutic targets for reversing ED (132). In summary, a thorough understanding of the gene and protein expression profiles specific to endothelial cells is vital for clarifying the molecular mechanisms behind ED and for developing effective therapeutic strategies.

Non-coding RNAs (ncRNAs), particularly microRNAs (miRNAs) and long non-coding RNAs (lncRNAs), have emerged as essential regulators of gene expression in various biological processes, including endothelial function. These ncRNAs influence the expression of target genes that are crucial for endothelial cell proliferation, apoptosis, and inflammatory responses, thereby affecting vascular homeostasis and the onset of ED. For instance, miR-126 has been demonstrated to enhance the expression of eNOS and promote the survival of endothelial cells, while its downregulation correlates with increased oxidative stress and inflammation within these cells (133). Moreover, lncRNAs such as ANRIL have been shown to play a role in regulating endothelial cell function by modulating the expression of genes associated with inflammatory pathways and vascular remodeling (134). The dysregulation of these ncRNAs has been associated with a variety of cardiovascular diseases, emphasizing their potential as both biomarkers and therapeutic targets. Additionally, the interaction between ncRNAs and signaling pathways, such as the Notch and Wnt pathways, highlights the intricate nature of gene regulation in endothelial cells and the possibility for targeted interventions aimed at restoring endothelial function (135). As research progresses in uncovering the roles of ncRNAs in endothelial biology, their therapeutic potential in preventing and treating diseases related to ED appears increasingly promising.

### 2.7 Treatment strategies and future perspectives for ED

Treatment of ED is most effective when aligned with dominant mechanistic modules, including restoration of NO signaling, suppression of pathological ROS sources, attenuation of endothelial inflammatory activation, stabilization of barrier integrity, and prevention of prothrombotic remodeling and EndMT-driven fibrosis. Conventional cardiometabolic therapies often improve endothelial phenotypes indirectly by controlling upstream drivers, whereas emerging strategies aim to enhance endothelial repair or directly modulate molecular pathways. A phenotype-guided approach may help match therapies to patient subgroups and define endpoints for monitoring response.

#### 2.7.1 Traditional drug therapy

Antihypertensive medications, particularly angiotensin-converting enzyme inhibitors (ACEIs), play a crucial role in managing ED, especially in patients with hypertension. For example, perindopril, a widely recognized ACEI, has been shown to improve endothelial function by reducing inflammatory markers like E-selectin and endothelin-1, which are often elevated in individuals with high blood pressure. These medications work by inhibiting the renin-angiotensin system, leading to vasodilation and lower blood pressure, which alleviates the mechanical stress on endothelial cells. Studies indicate that long-term use of ACEIs can significantly enhance endothelial function, as evidenced by improved flow-mediated dilation and reduced arterial stiffness. Moreover, the advantages of ACEIs extend beyond mere blood pressure control; they also exhibit anti-inflammatory and antioxidant properties that are vital in mitigating oxidative stress, a key contributor to ED. These findings suggest that ACEIs may be beneficial not only for patients with hypertension but also for those with various cardiovascular risk factors, highlighting their importance in a comprehensive strategy for preserving endothelial health (136, 137).

Lipid-lowering agents, especially statins, have become vital in managing ED linked to dyslipidemia. Statins not only lower low-density lipoprotein cholesterol levels but also enhance the activity of eNOS, which boosts NO production and improves vascular relaxation. This combined effect aids in restoring endothelial function and lowering the risk of cardiovascular events. Additionally, recent research has shown that statins can have anti-inflammatory effects by decreasing the expression of adhesion molecules and inflammatory cytokines, which are often elevated in cases of ED. Likewise, antidiabetic medications, particularly sodium-glucose cotransporter-2 inhibitors and glucagon-like peptide-1 receptor agonists, have demonstrated potential in enhancing endothelial function among diabetic patients. These medications not only improve blood sugar control but are also linked to positive cardiovascular outcomes, likely through mechanisms that reduce oxidative stress and inflammation. Therefore, incorporating both lipid-lowering and antidiabetic therapies into treatment plans for patients with ED is essential, as these medications target various pathways that contribute to vascular health (138).

The increasing recognition of the role of anti-inflammatory and antioxidant drugs in managing ED highlights their importance, especially considering the significant impact of inflammation and oxidative stress on cardiovascular diseases. Non-steroidal anti-inflammatory drugs and specific anti-inflammatory agents like canakinumab have shown promise in lowering systemic inflammation, which is often linked to ED. By focusing on inflammatory pathways, these medications can aid in restoring the integrity and function of the endothelium. Moreover, antioxidants such as vitamin E and flavonoids from natural sources, including those found in traditional Chinese medicine, have been investigated for their capacity to neutralize ROS and mitigate oxidative stress. For example, compounds like berberine and curcumin have been found to protect endothelial cells by increasing NO bioavailability and decreasing oxidative damage. The therapeutic approach of combining anti-inflammatory and antioxidant treatments may yield a synergistic effect, providing a comprehensive strategy to enhance endothelial function and lower the risk of cardiovascular complications. Continued research into the effectiveness and mechanisms of these agents will be essential for developing targeted therapies aimed at addressing ED (139, 140).

#### 2.7.2 Emerging therapeutic approaches

EPCs and stem cell therapies have emerged as promising strategies for treating ED, particularly in cardiovascular diseases. EPCs, which originate from the bone marrow, are crucial for repairing and regenerating damaged endothelium. Recent studies emphasize the potential of administering EPCs to enhance endothelial repair mechanisms, thereby improving vascular function. For example, clinical trials have shown that infusing autologous EPCs can lead to significant improvements in endothelial function and vascular health in patients with conditions such as coronary artery disease and peripheral artery disease (104). Furthermore, advancements in stem cell technology, particularly the use of induced pluripotent stem cells (iPSCs), have opened new avenues for generating endothelial cells for therapeutic applications. These iPSCs can be differentiated into endothelial cells, which can then be used to repair damaged blood vessels or create vascular grafts. The ability to produce patient-specific endothelial cells from iPSCs not only enhances the potential for personalized medicine but also addresses the shortage of donor tissues for transplantation. Additionally, ongoing research is focused on optimizing the conditions for EPC expansion and enhancing their functional capacity through genetic modification or the use of bioactive compounds, further solidifying their role in endothelial therapy (114).

Gene therapy represents a transformative approach in managing ED by directly targeting the underlying genetic and molecular mechanisms. Recent advancements have concentrated on delivering therapeutic genes that can enhance endothelial function or inhibit pathways leading to endothelial injury. For instance, delivering genes that encode eNOS has shown promise in restoring nitric oxide production, which is critical for maintaining vascular health. Additionally, ncRNAs, particularly miRNAs and lncRNAs, have emerged as key regulators of endothelial function and dysfunction. These molecules can modulate various signaling pathways involved in inflammation, oxidative stress, and apoptosis, all of which are pivotal in the pathogenesis of ED. Targeting specific miRNAs that promote endothelial repair or inhibit pro-inflammatory responses has been demonstrated to improve endothelial function in preclinical models (141). Furthermore, the development of RNA-based therapeutics, such as antagomirs or miRNA mimics, offers a novel strategy to manipulate endothelial cell behavior and enhance their regenerative capacity. As the field of gene therapy and ncRNAs research continues to evolve, the potential for these approaches to provide effective treatments for ED is becoming increasingly evident (142).

Small molecule activators have emerged as promising therapeutic agents for addressing ED, with sirtuin 1 (SIRT1) activators such as SRT1720 leading the charge in research efforts. SIRT1, a member of the sirtuin protein family, plays a vital role in maintaining endothelial function by influencing oxidative stress, inflammation, and apoptosis. When SIRT1 is activated, it has been shown to boost nitric oxide production, enhance the survival of endothelial cells, and promote angiogenesis, effectively counteracting the harmful effects associated with ED. Recent studies indicate that SRT1720 not only activates SIRT1 but also improves endothelial function across various animal models of cardiovascular disease (143). This compound has demonstrated the ability to lower oxidative stress and inflammation in endothelial cells, which contributes to better vascular reactivity and a decrease in atherosclerotic plaque formation. Furthermore, SRT1720's potential to improve metabolic health and lower the risk of cardiovascular events positions it as a strong candidate for further clinical development. As research continues, the investigation of small molecule activators like SRT1720 may lead to innovative pharmacological strategies aimed at restoring endothelial function and preventing the advancement of cardiovascular diseases (144).

#### 2.7.3 Lifestyle interventions and non-pharmacological treatment

Lifestyle interventions are essential for managing ED, a precursor to various cardiovascular diseases. Engaging in regular physical activity has been shown to improve endothelial function by enhancing nitric oxide bioavailability, reducing oxidative stress, and promoting vascular remodeling. Aerobic exercise, in particular, can lead to notable enhancements in endothelial health, as demonstrated by improved FMD measurements (145). Additionally, dietary changes, such as adopting a Mediterranean diet rich in fruits, vegetables, whole grains, and healthy fats, have been linked to better endothelial function and lower inflammation levels (146). Quitting smoking is another critical aspect of lifestyle intervention, as tobacco smoke is a significant risk factor for ED due to its oxidative effects and disruption of nitric oxide signaling. Research indicates that stopping smoking can result in considerable improvements in endothelial function, although the timeline for these benefits may differ from person to person (147). Together, these lifestyle modifications not only reduce the risk of developing cardiovascular diseases but also enhance overall metabolic health, addressing multiple risk factors related to ED.

Weight management plays a crucial role in preventing and treating ED, especially among individuals with obesity and metabolic syndrome. Excess body weight is associated with increased fat tissue, which can lead to systemic inflammation and damage to the endothelium. Effective strategies for weight loss, such as reducing caloric intake, increasing physical activity, and making behavioral changes, have shown significant benefits in enhancing endothelial function and lowering cardiovascular risk (148). For example, losing just 5%–10% of body weight can result in notable improvements in key metabolic indicators, including blood pressure, lipid levels, and insulin sensitivity, all of which are vital for maintaining healthy endothelial function (145). Furthermore, lifestyle changes that combine dietary adjustments with regular exercise have proven to be more effective than medications alone in achieving metabolic control and improving endothelial health (149). By integrating these approaches, individuals not only manage their weight more effectively but also adopt a comprehensive strategy for cardiovascular wellness, highlighting the critical role of lifestyle modifications in counteracting the negative impacts of obesity on endothelial function.

#### 2.7.4 Future research directions

The future of managing ED is centered on developing individualized treatment strategies and integrating precision medicine. This approach focuses on customizing therapies according to each patient's unique genetic, environmental, and lifestyle factors. Recent studies have underscored the significance of genetic predispositions in the risk of developing ED, particularly in conditions such as type 2 diabetes and cardiovascular diseases (22). By utilizing genomic data and biomarkers, healthcare providers can pinpoint patients who are most likely to benefit from specific therapeutic interventions, thereby enhancing treatment effectiveness. Additionally, precision medicine facilitates the inclusion of lifestyle modifications, like dietary changes and exercise programs, which have been proven to improve endothelial function (150). As research progresses in uncovering the molecular mechanisms behind ED, the opportunity to create targeted therapies that address these pathways will grow, ultimately leading to more effective and personalized treatment options for patients dealing with cardiovascular and metabolic diseases.

The complexity of ED requires a multi-target approach in therapy, as treatments using a single agent often do not adequately address the diverse nature of the condition. Combination therapies that target various pathways involved in ED could lead to better treatment outcomes. For example, recent studies have shown that pairing antihypertensive medications with agents that enhance endothelial function, such as statins or angiotensin receptor blockers, can produce synergistic effects (120). Furthermore, incorporating dietary bioactive compounds from fruits and vegetables alongside pharmacological treatments may further improve endothelial health by lowering oxidative stress and inflammation (150). Future research should aim to identify the best combinations of existing therapies and investigate new agents that can address multiple facets of ED, ultimately enhancing patient outcomes and alleviating the burden of cardiovascular diseases.

To enhance the understanding and management of ED, there is an urgent need for long-term clinical trials that evaluate the effectiveness of new therapeutic strategies and validate novel biomarkers. Recent research has identified several promising biomarkers linked to ED, such as circulating endothelial cells and microparticles, which could provide valuable insights into disease progression and treatment response (151). However, the clinical usefulness of these biomarkers remains to be confirmed. Longitudinal studies that monitor changes in these biomarkers alongside clinical outcomes will be crucial for determining their predictive value. Additionally, incorporating advanced imaging techniques and non-invasive assessments of endothelial function may improve our ability to track treatment effects and disease progression in real-time (28). By establishing reliable biomarkers and conducting thorough clinical trials, researchers can develop more effective management strategies for patients with ED, ultimately enhancing cardiovascular health outcomes on a broader scale.

## 3 Conclusions

ED is not a single entity but a set of inter-related, clinically measurable phenotypes that arise from a limited number of reinforcing molecular modules. Across cardiometabolic and cerebrovascular disease, reduced NO bioavailability and ROS excess form a central vicious cycle that integrates with inflammatory signaling (NF-κB/JAK/STAT, inflammasome activation, and senescence-associated programs) to drive endothelial activation, permeability, and prothrombotic remodeling. When these stressors persist, EndMT and other remodeling programs translate functional ED into structural fibrosis and vascular stiffness. Organizing ED by dominant phenotype (vasomotor dysfunction, barrier failure, inflammatory activation, EndMT/fibrosis) helps align biomarkers and functional/imaging tests with mechanism, improves risk stratification, and clarifies why therapies that restore NO signaling and suppress oxidative/inflammatory loops often provide broad vascular benefit. Future progress will depend on standardized biomarker panels and endpoints, longitudinal studies linking phenotype shifts to outcomes, and mechanism-guided trials that match interventions to the dominant ED module in each patient population.
